# The impact of public performance reporting on cancer elective surgery waiting times: a data linkage study

**DOI:** 10.1186/s12913-021-06132-w

**Published:** 2021-02-08

**Authors:** Khic-Houy Prang, Rachel Canaway, Marie Bismark, David Dunt, Julie A. Miller, Margaret Kelaher

**Affiliations:** 1 Centre for Health Policy, Melbourne School of Population and Global Health, The University of Melbourne, Melbourne, Victoria Australia; 2 Endocrine Surgery Unit, The Royal Melbourne Hospital, Melbourne, Victoria Australia; 3 Department of Surgery, Melbourne Medical School, The University of Melbourne, Melbourne, Victoria Australia

**Keywords:** Public reporting, Waiting times, Cancer, Surgery, Data linkage

## Abstract

**Background:**

Excessive waiting times for cancer elective surgery are a concern in publicly funded healthcare systems. Several countries including Australia have introduced healthcare reforms involving time-based targets and public performance reporting (PPR) of hospital data. However, there is mixed evidence of their benefits. We sought to examine the impact of targets and PPR of cancer elective surgery waiting times on access to breast, bowel and lung cancer elective surgery.

**Methods:**

We analysed routinely-collected linked data on admissions and waiting times for patients aged 15 years or over (*n* = 199,885) who underwent cancer surgery in a public hospital in Victoria, Australia over a 10-year period. We conducted difference-in-differences analyses to compare waiting times before (2006–07 to 2011–12) and after (2012–13 to 2015–16) the introduction of PPR in meeting these targets.

**Results:**

Across all cancer types, urgent patients were all treated within 30 days before and after PPR. Following PPR, there was a slight increase in the mean waiting times across all cancer types and urgency categories. Patients with lung cancer waited on average two and half days longer for treatment and patients with breast cancer waited on average half-a-day less. There was no effect of PPR on waiting times for patients with bowel cancer across urgency categories.

**Conclusions:**

Our findings suggest that time-based targets and PPR had minimal impact on surgical waiting times. This may be due to reasonable waiting times prior to PPR, improved efficiency being masked by 20% growth in the population, lack of public knowledge that waiting times are publicly reported, or lack of real-time reporting to drive behavioural change. The use of generic elective surgery recommended waiting time measures for cancer is discussed.

**Supplementary Information:**

The online version contains supplementary material available at 10.1186/s12913-021-06132-w.

## Background

Effective cancer care relies on timely access to treatment. For patients, delays in accessing needed treatment may worsen health outcomes, impair quality of life, induce anxiety and distress, and prolong sick leave and loss of income [1, 2]. For the public, delays in access can exacerbate health inequities, and lead to media and political pressure to address barriers to care [3, 4]. To improve the timeliness of elective surgery for cancer and other conditions, many countries have introduced healthcare reforms involving national targets and performance measures [5]. Previous studies have shown that patients with cancer support public reporting of care quality, timeliness, and patient satisfaction/experience [6, 7].

Waiting time for elective surgery is one of the most common measures of healthcare system performance. To improve the quality of hospital services, several countries publish national waiting times on websites, by specialties and procedures, using hospital administrative data [8]. Some countries, such as England, report at hospital and surgeon-levels and publish patient narratives [9, 10]. Public performance reporting (PPR) of hospital data is thought to improve quality of care by informing patients’ choice of provider, or by prompting organisations to address areas of underperformance [11]. Waiting times are generally measured as the time between the specialist placing the patient on the waiting list and the date of surgery. The wait between a general practitioner’s (GP) referral and the initial specialist appointment is not included, giving rise to a ‘hidden’ waiting list [12]. To better capture waiting times across the whole patient journey, some Nordic countries seek to measure the time between a GP’s referral and the date of surgery [13].

International evidence suggests that elective surgery targets can reduce waiting times and improve access over time [14]. The evidence is stronger in England [15, 16]. Ambitious waiting time targets with rewards and sanctions, increased funding and robust performance management, were cited as key factors contributing to the English success [14, 17]. Despite the reduction in waiting times, there was variation in waiting times between specialties, operative procedures and hospitals [18]. For PPR, there was a lack of evidence to suggest that publishing elective surgery waiting times motivated changes in providers’ behaviour to reduce waiting times or encouraged patients to choose providers based on PPR [19]. Targets and PPR had unintended consequences such as increased pressure on clinicians [20] and gaming of performance data [19, 21].

Previous research on waiting times has focused on certain specialities or all elective surgery [22–24]. Few studies have addressed delays in accessing cancer care [1, 2], so the impact of targets and published waiting times for elective surgery for cancer remains uncertain. Given the significant burden of morbidity and mortality associated with cancer [25], and concerns about the fragmentation of cancer care, some countries have developed specific strategies for improving access to cancer care [26]. These include collecting and monitoring waiting times from referral to first treatment for cancer. For example, England introduced targets for maximum waiting times across the cancer care pathway: a specialist appointment within 14 days of GP’s referral (93% target); first treatment within 31 days of diagnosis (96% target); and first treatment within 62 days of GP’s referral (85% target) [27]. There has been limited exploration of these issues in other international contexts including Australia.

In 2011, the Australian government introduced national elective surgery targets and PPR of hospital data, as part of a suite of national healthcare reforms to improve health outcomes. The objectives of the reforms were to optimise hospital care quality and timeliness for Australians [28]. State and Territories governments were offered financial rewards if certain national elective surgery targets were reached [29]. Implementation of the 2011 reforms were the responsibility of States and Territories, with the Commonwealth government responsible for PPR. The national elective surgery targets, underpinned by the Performance and Accountability Framework [30], included: elective surgery waiting times by urgency category (i.e. urgent - within 30 days, semi-urgent - within 90 days and non-urgent – within 12 months) and waiting times for cancer care. The MyHospitals website was established in 2010. All public hospitals were mandated to publicly report their performance on the MyHospitals website [31]; PPR is voluntary for private hospitals. The indicators publicly reported for breast, bowel and lung cancer at the time of the study included elective surgery waiting times by urgency and the number of patients treated within recommended elective surgery waiting times by urgency.

Victoria is the second most populous state in Australia; during the study period its population grew from 5.13 to 6.24 million – an increase of over 17% [32]. Australia has a universal healthcare system publicly funded through the Medicare scheme [33], including free access to treatment in public hospitals. A typical treatment pathway for cancer involves initial assessment by a GP or emergency department (ED) physician followed by referral to a specialist in either the public or private healthcare sectors. Acute conditions (such as a bowel cancer causing bowel obstruction) are treated immediately. Otherwise, patients are booked for ‘elective’ treatment or placed on a waiting list. Waiting times for publicly funded healthcare services arise when the demand for services exceeds the available supply. Population growth, an ageing population, increasing prevalence of chronic disease, funding constraints, and workforce shortages all contribute to this imbalance [34]. In the absence of price-rationing within the public system, waiting lists based on clinical need act as a form of non-price rationing to balance the demand for, and the supply of, health services. Around 40% of Australians choose to purchase private healthcare, or self-fund private care, which enables them to “skip the queue” in the public system by accessing elective surgery in private hospitals [35].

To our knowledge, our study is the first to examine the impact of the introduced targets and of PPR on cancer surgery waiting times in Victoria, Australia, principally in the public hospital system. Linkage of hospital admissions and elective surgery waiting time data provided an opportunity to examine whether the introduction of targets and PPR led to improvement in cancer elective surgery access during the 2006–2016 period, as measured by cancer waiting times indicators reported on the MyHospitals website. We sought to address the following research question: Does the introduction of PPR reduce waiting times for cancer types in which elective surgery waiting times are publicly reported, as compared to other cancer types in which elective surgery waiting times are not publicly reported?

## Methods

### Study design

The study involved a quasi-experimental research design using linked hospital admissions and elective surgery waiting time data to understand the effect of targets and PPR on cancer elective surgery waiting times. The pre-PPR period was defined as 2006–07 through 2010–11, and the post-PPR period as 2011–12 through to 2015–16.

### Data sources

#### Victorian Admitted Episodes Dataset

The Department of Health and Human Services mandates all Victorian hospitals to report patient admission activity to the Victorian Admitted Episodes Dataset (VAED) under the Health Services Act 1988 [36]. Data collection began in 1979 and continues to the present day with some changes to meet updated national reporting requirements. The VAED includes demographic and clinical information for each admitted episode of patient care, with clinical information coded according to the International Statistical Classification of Diseases and Related Health Problems, Tenth Revision, Australian Modification and the Australian Classification of Health Interventions [37].

#### The Elective Surgery Information System

The Elective Surgery Information System (ESIS) was introduced in 1997 to monitor access to elective surgery. Victorian public hospitals are required to provide episode-level elective surgery waiting list information to the Department of Health and Human Services. ESIS includes demographic, waiting times and other characteristics of elective surgery for each episode of patient care. Clinical information such as diagnosis are not captured in ESIS, and therefore linkage to VAED is required to identify patients with cancer. Episode-level data was considered an independent observation as multiple waiting list entries for a patient are acceptable if the procedures are independent of each other. Over the 10-year period, 80% of patients (*n* = 120,920) had one operation and 20% (*n* = 30,511) had multiple operations for cancer.

### Inclusion and exclusion criteria

We selected all public hospital discharge episodes for persons aged 15 years or more, admitted for bowel, breast, lung or other cancer elective surgery between July 2006 and June 2016. Principal cancer diagnosis and procedures were identified using the diagnosis and procedures codes (Additional file 1: Appendix 1). Mapping of the diagnosis (2006 to 2016 edition) and procedure (5th to 9th edition) codes was conducted to ensure consistent coding over time. ‘Admission type’ and ‘Diagnosis Related Groups type’ were limited, respectively, to ‘admission from the waiting list’ and ‘surgical’. We excluded admissions classified as clinical urgency category 3 (i.e. admission within 365 days) due to the small number of cases. Waiting times for patients who were yet to receive their surgery at the end of the study period were not included.

### Outcome

The outcome of interest was total waiting time for cancer elective surgery, defined as the number of days between being placed on a waiting list for surgery and the date of admission for that surgery. This does not include the waiting time between a GP’s referral and the initial specialist appointment.

### Data linkage

Deterministic data linkage of VAED and ESIS records for the period from 2006 to 07 to 2015–16 was undertaken by the Centre for Victorian Data Linkage (CVDL) [38]. Cases were extracted from the VAED using the inclusion criteria and then linked to ESIS using Medicare number and suffix, date of birth, sex, and hospital unit record number. The de-identified linked dataset was provided for analysis by CVDL.

### Statistical analysis

We compared demographic characteristics of patients with breast, bowel, lung or other cancer before and after PPR. A total treatment group was created by combining patients with breast, bowel or lung cancer together. We conducted difference-in-differences (DID) analyses to compare the change in total waiting times after the introduction of PPR for each cancer type and the total treatment group. DID analysis is a commonly used empirical technique in quasi-experimental studies to evaluate the impact of a policy as it measures the change in an outcome before and after an intervention between treatment and control groups, then subtracts one from the other to see the ‘difference in the differences’ [39]. DID analysis is usually implemented as an interaction term between intervention and time in a regression model. An assumption of DID analysis is parallel trends, in which the pre-intervention trends in outcomes are the same between the treatment and control groups. Visual inspections of the pre-treatment trends for the treatment and control groups were conducted (Additional file 1: Appendix 2). Breast, bowel and lung cancer were considered the treatment group because their waiting time indicators were publicly reported on the MyHospitals website, and other cancers were the control group (their waiting time indicators were not publicly reported). Each model was adjusted for sex, age group, marital status, preferred language, patient region, patient type, urgency, surgical specialty, length of stay, and hospital peer group. A hospital peer group consists of similar hospitals based on shared characteristics such as hospital size, service provision and geographical location, thus enabling hospitals to be compared to other similar hospitals to ensure valid comparisons are made [40]. A *p*-value of less than 0.05 was considered statistically significant in all analyses. Data analyses were conducted using Stata version 14.

### Ethical considerations

Ethical approval for the study was granted by the Melbourne School of Population and Global Health Human Ethics Advisory Group, The University of Melbourne.

## Results

### Patient characteristics

Descriptive statistics of patient characteristics by cancer types before and after PPR are presented in Table 1. Over the 10-year period there were 199,885 cancer elective surgeries. Of those, 4.6% (*n* = 9104) surgeries were for bowel, 8.5% (*n* = 16,926) for breast, and 1.5% (*n* = 3037) for lung cancers. Males accounted for the majority of bowel and lung cancer surgeries. Bowel and lung cancer surgeries were most common among those aged 70–74 years, while breast cancer surgeries commonly occurred among those aged 50–54 years. The majority attended a metropolitan area hospital. Patients with bowel and breast cancers were commonly treated in large hospitals (public acute group A hospitals) and patients with lung cancer in major referral hospitals. The mean length of stay was the shortest for breast cancers, followed by lung and bowel cancers.

**Table 1:** Demographic characteristics of patients by cancer types before and after public performance reporting (*N* = 199,885)

	Bowel cancer ***n*** = 9104		Breast cancer ***n*** = 16,926		Lung cancer ***n*** = 3037		Bowel, breast and lung cancer ***n*** = 29,067		Other cancer ***n*** = 170,818	
	Pre-PPR ***n*** = 4664	Post-PPR ***n*** = 4440	Pre-PPR ***n*** = 7197	Post-PPR ***n*** = 9729	Pre-PPR ***n*** = 1205	Post-PPR ***n*** = 1832	Pre-PPR ***n*** = 13,066	Post-PPR ***n*** = 16,001	Pre-PPR ***n*** = 77,110	Post-PPR ***n*** = 93,708
**Sex**										
*Male*	2680 (57.5%)	2580 (58.1%)	55 (0.8%)	78 (0.8%)	685 (56.8%)	984 (53.7%)	3420 (26.2%)	3642 (22.8%)	36,030 (46.7%)	44,062 (47.0%)
*Female*	1984 (42.5%)	1860 (41.9%)	7142 (99.2%)	9651 (99.2%)	520 (43.2%)	848 (46.3%)	9646 (73.8%)	12,359 (77.2%)	41,080 (53.3%)	49,646 (53.0%)
**Age group**										
*15–24*	15 (0.3%)	8 (0.2%)	13 (0.2%)	6 (0.1%)	2 (0.2%)	9 (0.5%)	30 (0.2%)	23 (0.1%)	3300 (4.3%)	3092 (3.3%)
*25–34*	34 (0.7%)	63 (1.4%)	182 (2.5%)	240 (2.5%)	8 (0.7%)	9 (0.5%)	224 (1.7%)	312 (2.0%)	6530 (8.5%)	7929 (8.5%)
*35–44*	124 (2.7%)	174 (3.9%)	874 (12.1%)	1006 (10.3%)	28 (2.3%)	28 (1.5%)	1026 (7.9%)	1208 (7.6%)	8559 (11.1%)	9725 (10.4%)
*45–54*	477 (10.2%)	497 (11.2%)	1793 (24.9%)	2347 (24.1%)	120 (10.0%)	147 (8.0%)	2390 (18.3%)	2991 (18.7%)	11,480 (14.9%)	13,870 (14.8%)
*55–64*	1001 (21.5%)	905 (20.4%)	1850 (25.7%)	2457 (25.3%)	310 (25.7%)	416 (22.7%)	3161 (24.1%)	3778 (23.6%)	13,516 (17.5%)	16,393 (17.5%)
*65–74*	1515 (32.5%)	1329 (29.9%)	1496 (20.8%)	2275 (23.4%)	474 (39.3%)	775 (42.3%)	3485 (26.7%)	4379 (27.4%)	15,217 (19.7%)	19,432 (20.7%)
*75–84*	1254 (26.9%)	1220 (27.5%)	788 (10.9%)	1108 (11.4%)	255 (21.2%)	426 (23.3%)	2297 (17.6%)	2754 (17.2%)	13,892 (18.0%)	16,907 (18.0%)
*85+*	244 (5.2%)	244 (5.5%)	201 (2.8%)	290 (3.0%)	8 (0.7%)	22 (1.2%)	453 (3.5%)	556 (3.5%)	4646 (6.0%)	6360 (6.8%)
**Marital status**										
*Never married*	475 (10.2%)	547 (12.3%)	938 (13.0%)	1369 (14.1%)	147 (12.2%)	261 (14.2%)	1560 (11.9%)	2177 (13.6%)	15,131 (19.6%)	18,929 (20.2%)
*Widowed/divorced/separated*	1158 (24.8%)	1025 (23.1%)	1871 (26.0%)	2446 (25.1%)	258 (21.4%)	441 (24.1%)	3287 (25.2%)	3912 (24.5%)	16,106 (20.9%)	18,761 (20.0%)
*Married/de facto*	3015 (64.6%)	2849 (64.2%)	4348 (60.4%)	5843 (60.1%)	795 (66.0%)	1115 (60.9%)	8158 (62.4%)	9807 (61.3%)	45,427 (58.9%)	55,420 (59.1%)
*Not stated*	16 (0.3%)	19 (0.4%)	40 (0.6%)	71 (0.7%)	5 (0.4%)	15 (0.8%)	61 (0.5%)	105 (0.7%)	446 (0.6%)	598 (0.6%)
**Preferred language**										
*English*	3953 (84.8%)	3795 (85.5%)	6383 (88.7%)	8644 (88.8%)	1053 (87.4%)	1603 (87.5%)	11,389 (87.2%)	14,042 (87.8%)	69,037 (89.5%)	84,286 (89.9%)
*Others*	711 (15.2%)	645 (14.5%)	814 (11.3%)	1085 (11.2%)	152 (12.6%)	229 (12.5%)	1677 (12.8%)	1959 (12.2%)	8073 (10.5%)	9422 (10.1%)
**Patient type**										
*Public*	4327 (92.8%)	4041 (91.0%)	6668 (92.6%)	8598 (88.4%)	1040 (86.3%)	1640 (89.5%)	12,035 (92.1%)	14,279 (89.2%)	69,091 (89.6%)	82,381 (87.9%)
*Private*	289 (6.2%)	365 (8.2%)	494 (6.9%)	1086 (11.2%)	140 (11.6%)	182 (9.9%)	923 (7.1%)	1633 (10.2%)	6702 (8.7%)	10,344 (11.0%)
*Others*^*a*^	48 (1.0%)	34 (0.8%)	35 (0.5%)	45 (0.5%)	25 (2.1%)	10 (0.5%)	108 (0.8%)	89 (0.6%)	1317 (1.7%)	983 (1.0%)
**Hospital region**										
*Metropolitan*	3488 (74.8%)	3280 (73.9%)	5593 (77.7%)	7461 (76.7%)	1067 (88.5%)	1561 (85.2%)	10,148 (77.7%)	12,302 (76.8%)	64,211 (83.3%)	77,345 (82.5%)
*Rural*	1176 (25.2%)	1160 (26.1%)	1604 (22.3%)	2268 (23.3%)	138 (11.5%)	271 (14.8%)	2918 (22.3%)	3699 (23.1%)	12,899 (16.7%)	16,363 (17.5%)
**Hospital peer groups [AIHW]**										
*Principal referral*^*b*^	1576 (33.8%)	1303 (29.3%)	2047 (28.4%)	2478 (25.5%)	865 (71.8%)	1382 (75.4%)	4488 (34.4%)	5163 (32.3%)	26,056 (33.8%)	28,914 (30.9%)
*Public acute group A*^*c*^	2901 (62.2%)	2918 (65.7%)	4218 (58.6%)	4979 (51.2%)	340 (28.2%)	423 (23.1%)	7459 (57.1%)	8320 (52.0%)	32,411 (42.0%)	36,773 (39.2%)
*Public acute group B*^*d*^	182 (3.9%)	93 (2.1%)	644 (8.9%)	855 (8.8%)	0 (0.0%)	0 (0.0%)	826 (6.3%)	948 (5.9%)	9524 (12.4%)	10,964 (11.7%)
*Public acute group C*^*e*^	0 (0.0%)	0 (0.0%)	166 (2.3%)	639 (6.6%)	0 (0.0%)	0 (0.0%)	166 (1.3%)	639 (4.0%)	2544 (3.3%)	4245 (4.5%)
*Other public acute specialised*	0 (0.0%)	119 (2.7%)	0 (0.0%)	397 (4.1%)	0 (0.0%)	27 (1.5%)	0 (0.0%)	543 (3.4%)	876 (1.1%)	4691 (5.0%)
*Women’s*	4 (0.1%)	6 (0.1%)	122 (1.7%)	380 (3.9%)	0 (0.0%)	0 (0.0%)	126 (0.9%)	386 (2.4%)	5442 (7.1%)	7525 (8.0%)
*Children’s*	1 (0.0%)	1 (0.0%)	0 (0.0%)	0 (0.0%)	0 (0.0%)	0 (0.0%)	1 (0.0%)	1 (0.0%)	105 (0.1%)	187 (0.2%)
*Other day procedure hospital*	0 (0.0%)	0 (0.0%)	0 (0.0%)	1 (0.0%)	0 (0.0%)	0 (0.0%)	0 (0.0%)	1 (0.0%)	152 (0.2%)	409 (0.4%)
**Length of stay**										
*Mean (standard deviation)*	11 (10)	10 (11)	3 (4)	4 (5)	10 (7)	9 (8)	7 (8)	6 (7)	3 (6)	3 (6)

*PPR* public performance reporting

^a^ Others include compensable, Department of Veterans’ Affairs and ineligible patients

^b^ Provide broad range of services, range of highly specialised service units, and very large patient volume, located in major cities

^c^ Provide wide range of services but not the breath of services provided by Principal referral hospital, comparatively large and located in major cities and regional areas

^d^ Do not have service profile of Principal referral and public acute group A, comparatively large, half located in major cities and half in inner/outer regional areas

^e^ Limited range of services, generally smaller and located in inner/outer regional areas

### Waiting times

The mean and median cancer elective surgery waiting times by urgency category and cancer types are presented in Table 2. Following PPR, there was a slight increase in the mean and median waiting times across all cancer types and urgency category. The largest increase in waiting times occurred in lung cancer.

**Table 2:** Cancer elective surgery waiting times (in days) by urgency category and cancer type

	Bowel cancer		Breast cancer		Lung cancer		Bowel, breast and lung cancer		Other cancer	
	Pre-PPR	Post-PPR	Pre-PPR	Post-PPR	Pre-PPR	Post-PPR	Pre-PPR	Post-PPR	Pre-PPR	Post-PPR
**All urgency**										
*Mean (SD)*	14 (15)	17 (14)	13 (12)	14 (14)	12 (11)	17 (12)	13 (13)	15 (14)	33 (45)	35 (48)
*Median (IQR)*	13 (7–20)	15 (8–22)	11 (6–17)	12 (7–19)	9 (5–18)	15 (8–23)	12 (6–18)	13 (7–20)	19 (8–38)	20 (11–38)
**Urgency (30 days)**										
*Mean (SD)*	13 (8)	14 (8)	11 (7)	12 (7)	10 (8)	15 (8)	12 (7)	13 (7)	12 (8)	14 (8)
*Median (IQR)*	13 (7–19)	14 (8–21)	10 (6–16)	12 (7–18)	8 (5–15)	14 (7–21)	11 (6–17)	13 (7–19)	12 (6–19)	14 (7–20)
**Urgency (90 days)**										
*Mean (SD)*	37 (44)	42 (27)	33 (35)	42 (48)	25 (17)	33 (21)	33 (36)	39 (36)	59 (57)	64 (63)
*Median (IQR)*	33 (15–41)	36 (27–50)	25 (8–44)	32 (15–48)	23 (11–35)	33 (16–42)	28 (11–41)	34 (19–48)	44 (24–77)	47 (27–81)

*PPR* public performance reporting, *SD* standard deviation, *IQR* interquartile range

### Treatment within recommended time

The proportion of discharged patients treated within recommended elective surgery waiting times by urgency category and cancer types are presented in Table 3. Across all cancer types, patients classified as urgent were all treated within 30 days before and after PPR. Similarly, the majority of patients classified as semi-urgent were treated within 90 days for all cancer types; although there was a small decrease in the proportion of patients with bowel or breast cancer treated within 90 days following PPR. However, this was not statistically significant following the conduct of chi-square tests.

**Table 3:** Patients treated within recommended elective surgery waiting times by urgency category and cancer type

	Bowel cancer		Breast cancer		Lung cancer		Bowel, breast and lung cancer		Other cancer	
	Pre-PPR	Post-PPR	Pre-PPR	Post-PPR	Pre-PPR	Post-PPR	Pre-PPR	Post-PPR	Pre-PPR	Post-PPR
**Urgency (30 days)**										
*Yes*	4346 (99.9%)	3994 (99.9%)	6735 (100.0%)	9281 (100.0%)	1050 (100.0%)	1617 (100.0%)	12,131 (99.9%)	14,892 (99.9%)	42,875 (99.9%)	54,670 (99.8%)
*No*	3 (0.1%)	4 (0.1%)	1 (0.0%)	0 (0.0%)	0 (0.0%)	0 (0.0%)	4 (0.0%)	4 (0.0%)	62 (0.1%)	92 (0.2%)
**Urgency (90 days)**										
*Yes*	304 (96.5%)	420 (95.0%)	434 (94.1%)	407 (90.8%)	154 (99.4%)	213 (99.1%)	892 (95.8%)	1040 (94.1%)	27,895 (81.6%)	31,303 (80.4%)
*No*	11 (3.5%)	22 (5.0%)	27 (5.9%)	41 (9.2%)	1 (0.6%)	2 (0.9%)	39 (4.2%)	65 (5.9%)	6278 (18.4%)	7643 (19.6%)

*PPR* public performance reporting

### Difference-in-differences analyses

Table 4 presents the results of the DID models comparing the change in waiting times to treatment for breast, bowel and lung cancers, the total treatment group with other cancers, stratified by urgency category, and adjusted for sex, age group, marital status, preferred language, patient region, patient type, surgical specialty, length of stay and hospital peer group. There was an effect of PPR on waiting times to treatment for breast and lung cancers for urgent cases but not for semi-urgent cases*.* Patients with breast cancer waited on average half a day less for treatment than patients with other cancer types following PPR. In contrast, patients with lung cancer waited on average two and half days more for treatment than patients with other cancer types following PPR. There was no effect of PPR on waiting times to treatment for bowel cancer across urgency category. For the total treatment group, there was an effect of PPR on waiting times to treatment for urgent cases but not for semi-urgent cases. Patients with breast, bowel or lung cancer waited on average a quarter of a day less than patients with other cancer types following PPR.

**Table 4:** Adjusted difference-in-difference model regression results for effect of public performance reporting on cancer elective surgery waiting times stratified by urgency category

	Urgency (30 days)			Urgency (90 days)		
	β	SE	***p***-value	β	SE	***p***-value
Bowel cancer^a^	0.60	0.13	< 0.001	−19.66	3.43	< 0.001
Post PPR^b^	1.65	0.51	< 0.001	5.38	0.45	< 0.001
Post PPR*bowel cancer^c^	−0.23	0.18	0.20	0.27	4.40	0.95
N observations	106,046			73,876		
Breast cancer^a^	−0.76	0.11	< 0.001	−27.48	2.82	< 0.001
Post PPR^b^	1.67	0.05	< 0.001	5.41	0.45	< 0.001
Post PPR*breast cancer^c^	−0.51	0.13	< 0.001	5.23	3.97	0.19
N observations	113,716			74,028		
Lung cancer^a^	0.06	0.31	0.84	−8.12	5.83	0.16
Post PPR^b^	1.65	0.05	< 0.001	5.42	0.45	< 0.001
Post PPR*lung cancer^c^	2.50	0.31	< 0.001	−0.21	6.29	0.97
N observations	100,366			73,489		
Bowel, breast and lung cancer^a^	−0.24	0.09	< 0.01	−22.77	2.04	< 0.001
Post PPR^b^	1.69	0.05	< 0.001	5.36	0.44	< 0.001
Post PPR*bowel, breast and lung cancer^c^	−0.23	0.12	0.03	2.97	2.67	0.27
N observations	124,730			75,155		

All models adjusted for sex, age group, marital status, preferred language, patient region, patient type, surgical specialty, length of stay, AIHW hospital peer group

*SE* standard error, *PPR* public performance reporting

^a^ Difference between the two groups pre-intervention, with the control group (other cancer) as the reference group

^b^ Difference between pre and post-intervention in the control group (other cancer), with pre-intervention as the reference group

^c^ Difference in changes over time between the two groups

## Discussion

There are several possible explanations for the limited change in waiting times following PPR. First, lack of public knowledge that waiting times are publicly reported may have prevented the use of PPR for quality improvement. Previous studies have shown that patients and providers, including medical officers and GPs, are generally not aware of PPR data and are unclear what it is [41–44]. Second, the MyHospitals website does not provide “real-time” data and the data are not reported by diagnosis. The most recent time-period data published on the MyHospitals website were 2012–13 for cancer elective surgery waiting times and 2017–18 for elective surgery waiting times. Although the latest elective surgery waiting times were reported; data are provided by urgency category, specialty of surgeon, and intended procedure, but not diagnosis. This means that neither patients nor providers were able to access timely information about cancer-specific waiting times on the performance reporting website. As a result, there were limited opportunities for patients or providers to change their behaviour in response to such data.

Third, patients with breast, bowel or lung cancer waited on average 13 days for urgent care and 40 days for semi-urgent care, well below the recommended 30 and 90 days, respectively. The availability of relatively timely care within the Victorian jurisdiction means that pressure for change following PPR may have been less than in jurisdictions with unacceptably long waiting times. Notably, there was a slight increase in waiting times for patients with lung cancer following PPR which likely reflected the marked increase in lung cancer incidence rate among females in Victoria [45] and Australia [46]. This is likely to be attributed to the smoking pattern in the past decades (the prevalence of smoking in female peaked in the mid-1970s). Other factors which may explain the continued increase in lung cancer incidence rate include the changing composition of modern cigarettes and changes in age and size of the population.

Fourth, the population of Victoria grew by more than 1.1 million people during the study period. It is possible that the PPR led to improvements in efficiency within the healthcare system which were masked by the significant increase in demand associated with the rapidly growing population. As of 30th July 2020, there were over 56,000 patients on the waiting list across all urgency categories [47]. It was not possible to ascertain how many cases were cancer-related as ESIS records intended procedure and not patient diagnosis. Knowledge of diagnosis requires linkage to VAED following an admission to hospital [36]. As such, the data included only patients with cancer who were admitted to hospital for surgery and excluded those who may still be on the waiting list at the end of the study period or dropped out from the waiting list (e.g. surgery was no longer required, they passed away) during the study period. Further research is warranted to investigate the demand of cancer elective surgery and the capacity of the healthcare system to deliver timely cancer services.

Despite achieving the elective surgery waiting time targets, it is unclear whether the waiting times for cancer treatment are clinically appropriate given that they are prioritised using the same waiting list system as other elective surgeries such as hip replacement. Setting a generic waiting time for all cancer surgeries has been criticised for not considering the complexity of the disease, the phases of cancer care and the different treatment modalities of the various cancer types [48]. International guidelines in the UK for acceptable waiting times focus on the following phases of cancer care: days between GP’s referral and first specialist appointment; days between GP’s referral and diagnosis; days between decision to treat and start of treatment; and days between GP’s referral and start of treatment [27]. In Australia, Cancer Council Australia have introduced optimal cancer care pathways for 15 cancer types, with variation in recommended waiting times across cancer care phases [49]. There is an opportunity to capture and report waiting time indicators by diagnosis across the entire patient journey by using linked primary care and hospital data. This will help identify areas for improvement in care co-ordination, clinical practice and health services delivery at each stage of the cancer journey to be identified. Linkage of primary care and hospital data for cancer care is currently underway in Victoria, Australia, but collection of primary care data for linkage is not yet widespread [50–52].

Waiting times to elective surgery is one of the most common generic health service ‘quality’ measures publicly reported, alongside length-of-stay, complications and mortality. These measures are relatively simple to collect with the use of administrative data but provide limited insights into the quality of healthcare delivery. De-identified linkage of cancer registries with administrative data (e.g. electronic medical records) could provide a more complete picture of the quality of cancer care and health system performance [53]. Using these data, Spinks et al. [48] proposed collecting and reporting the following meaningful quality indicators across the cancer care continuum: outcomes (e.g. recovery, functional restoration, and survival); structure (e.g. physical facilities, nurse-to-patient ratios); process (e.g. screening, prevention, diagnosis and staging); efficiency (e.g. adherence to guidelines); cost of care (e.g. direct and indirect costs); and patients’ perceptions of care (e.g. satisfaction).

### Strengths and limitations

The study included state-wide population coverage of cancer elective surgery admissions over a period of 10 years. Despite the large time-period and a suitable comparator group, the findings should be interpreted in the context of several study limitations. A prerequisite of DID analysis is finding a control group for which the parallel trends assumption is met, in which the pre-intervention trends in outcomes are the same between the treatment and control groups. Ideally, the only difference between the two groups would be exposure to the policy. In practice, such a group may be difficult to find. The characteristics of the control group differ slightly from each of the intervention group but their trends in pre-treatment outcomes followed a similar trajectory. As such, selection bias may exist, and the results should be interpreted with caution.

The Australian healthcare reforms included several hospital and related care policies not limited to reducing waiting times for elective surgery and making service performance information publicly available [28]. As such, we were unable to disentangle the effect of confounding influences on cancer elective surgery waiting times, thus making it difficult to attribute the observed changes to a specific policy. Furthermore, it was unclear what quality improvement initiatives (if any) were implemented in the hospitals following the introduction of PPR that would drive or impede improvement in cancer elective surgery waiting times. Further research is required to investigate the causal pathways in which PPR influence waiting times via quality improvement processes.

There has been some evidence of manipulation of the elective surgery waiting list in Victoria, Australia [54, 55]. Patients classified as urgent or semi-urgent whose waiting times for surgery were approaching the target for their category were reclassified as “not ready for care – patient initiated”. This ensured that category waiting time targets were not exceeded and that the hospital met elective surgery key performance indicators [56, 57]. It is unclear how prevalent data manipulation is and whether this influenced our results.

The total elective surgery waiting times does not include the waiting time to see a specialist following a GP’s referral, which underestimates the total waiting times across the cancer care continuum, masking the true demands on the public hospital sector and the impact on public patients. There is no national administrative system in Australia that captures data on how many patients are waiting for these appointments, nor how long they have waited for them; although individual hospitals may collect such information in their internal database as referrals and appointments are dated and documented. Further research is warranted to capture the complete picture of waiting times for cancer elective surgery for policy makers to make fully informed decisions about public hospital service planning, delivery and resourcing.

Improved elective surgery waiting times would not necessarily represent an improvement in patient care; we did not have information on cancer tumour stage and clinical outcomes of patients. Future research is warranted to better understand the relationships between timeliness of care delivery and clinical outcomes. The study included public hospitals and generalisability to the private sector is unknown; however, it is expected that private patients treated in private hospitals would have shorter waiting times [58–60]. During the study period, the proportion of Australians with private health insurance increased from 9 million (44% of the population) to 11 million (47% of the population) [61]. Access to the Medicare Benefits Schedule data [62] or the National Hospital Morbidity Database [63] could provide insights into the number of cancer patients who underwent surgical treatment in the private sector. Medicare Benefits Schedule is a list of Medicare services subsidised by the Australian government which includes private patients in public or private hospitals who made a Medicare claim. The National Hospital Morbidity Database includes episode-level records from admitted patient morbidity data collection systems in Australian public and private hospitals. Further research is warranted to explore waiting times differences between public and private patients, and whether publicly reported waiting times in public hospitals influence cancer patients’ decision to seek treatment in the private sector.

## Conclusions

Our findings suggest that the introduction of elective surgery waiting time targets and PPR, which enable greater transparency of cancer elective surgery waiting times, had limited impact on patient waiting times in Victoria, Australia. Nonetheless, publicly reporting waiting times may still support patients with cancer to make an informed choice about their surgery. It will give patients the opportunity to pursue other avenues for surgery if made aware of the ‘true’ waiting times. This will be dependent on having ‘real-time’ dashboard/website, supplemented with relevant and meaningful quality indicators across the cancer care continuum for individual cancer types to inform patients’ choice.

## Supplementary Information

**Additional file 1: Appendix 1** Cancer diagnosis and procedure codes. **Appendix 2** Visual inspection of parallel trends in the pre-intervention period

## Data Availability

The data that support the findings of this study are available from CVDL but restrictions apply to the availability of these data, which were used under license for the current study, and so are not publicly available. Data are however available from CVDL for researchers who meet the criteria for access to confidential data. Information related to the process for requesting data is contained on the CVDL website: https://www2.health.vic.gov.au/about/reporting-planning-data/the-centre-for-victorian-data-linkage

## Abbreviations

CVDL: The Centre for Victorian Data Linkage
DID: Difference-in-Differences
ED: Emergency Department
ESIS: Elective Surgery Information System
GP: General Practitioner
PPR: Public Performance Reporting
VAED: Victorian Admitted Episodes Dataset
