# Supplementary material for: The impact of public performance reporting on cancer elective surgery waiting times: a data linkage study
Source: BMC Health Serv Res. 2021 Feb 8;21:129. doi: 10.1186/s12913-021-06132-w (PMC7871621; doi:10.1186/s12913-021-06132-w)
Supplement: Supplementary file 1 — Additional file 1: Appendix 1 Cancer diagnosis and procedure codes. Appendix 2 Visual inspection of parallel trends in the pre-intervention period [file 12913_2021_6132_MOESM1_ESM.docx]

**Appendix 1 Cancer diagnosis and procedure codes**

| **Cancer types** | **ICD-10 AM** | **ACHI** |
| --- | --- | --- |
| Bowel | C18, C19, C20 | 3200000, 3200001, 3200300, 3200301, 3200400, 3200401, 3200500, 3200501, 3200600, 3200601, 3200900, 3201200, 3201500, 3202400, 3202500, 3202600, 3202800, 3203000, 3203900, 3204700, 3205100, 3205101, 3206000, 3209900, 3211200, 9220800 |
| Breast | C50 | 3030000, 3033200, 3033500, 3033600, 3150000, 3151500, 3151800, 3151801, 3152400, 3152401 |
| Lung | C34 | 3843800, 3843801, 3843802, 3844000, 3844001, 3844100, 3844101, 9016900 |
| Other | C00-C97, D00-D48 (excluding bowel, breast and lung cancer) |  |

ICD-10 AM International Statistical Classification of Diseases and Related Health Problems, Tenth Revision, Australian Modification; ACHI Australian Classification of Health Interventions

**Appendix 2 Visual inspection of parallel trends in the pre-intervention period**
